# Gonnorrhœal Ophthalmia

**Published:** 1885-09

**Authors:** T. D. Wooten

**Affiliations:** Austin, Texas


### GONORRHŒAL OPHTHALMIA.

*By. T. D. Wooten, M. D. Austin, Texas.*

Read before Travis County Medical Society, and by resolution of that Society contributed to Daniel's Medical Journal.

GONORRHŒAL OPHTHALMIA is the most virulent, dangerous and destructive disease of the eye. It is caused by gonorrhœal matter coming in contact with the conjunctiva. This disease, so dangerous to vision, is very instructive in its relation to the specific nature of gonorrhœa. Catarrh is one of the most frequent maladies, and when inoculated on the conjunctiva, merely produces, at most, a slight and transitory catarrh. So in trachoma, inoculation only gives rise to trachoma; so it is when a gonorrhœal pus-cell is applied to the conjunctiva, it excites the well-known and dangerous gonorrhœal ophthalmia.

The course of the disease is somewhat characteristic of the specific nature of its origin. There is a stage of inoculation varying from a few hours to a few days; during this period the gonorrhœal germs did not exist in sufficient qnantity to be pathological, until, by sufficient multiplication to set up inflammation, with its specific metamorphic change upon the cells of the mucous membrane. What the precise nature of this poison or contagion is,I believe the present knowledge of the subject does not definitely enable me to state. Nor is it the province of this paper to enter into a discussion upon this line of the sub ject. It is in the highest degree probable, that there is, in gonorrhœal pus, a specific contagious element, gonococcus, or some fungoid specific germ that, not only sets up, but maintains this contagious inflammation. The infectious property, belonging specifically to gonorrhœal pus, when brought in contact with the conjunctiva, consist not only in producing the tissue changes incident to the ordinary process of inflammation, such as, first, local hyperæmia with its consequent manifestations of heat, redness, etc.; second, tissue-metamorphosis, etc., but has, in addition, the inherent quality of intensifying, rendering greater activity to the process; especially does it stimulate to great activity in metamorphic tissue change. As shown by the excess sive suppurative action attendant upon the disease,I believe the present accepted idea of the change, wrought upon the tissues by inflammation, is that the change consists in reducing them to the embryonic state. The normal tissues are normally composed of cells and intermediate substance. By inflammatory changes these are converted into ameoboid cells. The subdivided ameoboid cells are called pus-corpuscles. It is accordingly the tissue itself formed into pus-corpuscels. Suppuration presupposes loss of tissue. Pus, indeed, is the product of the splitting up, or proliferation, of infiltrated cells. Suppuration is, therefore, a transformation, or the metamorphosis of tissue. Suppuration and tissue genesis may coexist. A moderate production of normal pus on the surface of an ulcer, offers no impediment to recovery. When, however, the suppuration is too profuse, it not only prevents tissue genesis, but it disintegrates newly formed tissue. I have thought it necessary thus to epitomize some of the phenomena attendant upon ordinary inflammation and suppuration, in order to a better understanding of the manner of the destructive suppurative action attendant upon gonorrhœal inflammation; and the more fully convey the idea that an atom of gonorrhœal pus has the specific effect of inciting a virulent form of suppurative inflammation destructive in itself. But that in addition, it possesses the inherent property of imparting its infection to each atom of pus metamorphosis by that inflammatory action, and that each pus cell is barbed by an infectious germ, that acts as a local irritant, and reinoculates the tissues by contact, and has in fact, a direct dissolving and disintegrating effect upon the cornea and other tissues of the eye.

It is mentioned by very high and creditable authority that the inoculation of the conjunctiva with ordinary pus will produce an inflammation closely allied, if not identical, with gonorrhœal inflammation. I believe this to be a mistake; ordinary pus may produce an inflammatory action in the conjunctiva. It utterly fails to produce gonorrhœal inflammation. I have had frequent occasion to perform surgical operations in, and about the eyes, and have seen the conjunctiva covered in pus for several days without developing an inflammation at all allied in virulency with gonorrhœal ophthalmia, which is always due to inoculation, and traceable to the source of gonorrhœal infection. It has a period of inoculation, but much shorter than in the urethra. This may depend upon the fact that the conjunctiva affords a better culture for the infection, the muco-purulent discharge is increased with greater rapidity and in greater quantities. The morbific process is more destructive to the tissues of the eye than to those of the urethra. This may be due in part to the want of a free escape of the pus. The germs of conjunctiva being retained, and have the opportunity of more rapid propegation. Whereas in the urethra they are constantly being disturbed,and the discharge continually washed out by the frequent passage of urine. The eye, being more sensitive, more blood is invited to feed the inflammatory action. At any rate, a very high and active grade of inflammation rapidly ensues after inoculation. In a few hours a muco-purulent discharge sets in. The ocular portion of the conjunctiva becomes infiltrated with serum. This thickening rapidly increases, and forms chemosis,which is a very marked symptom in gonorrhœal cases. At the same time the lids become red and swollen; the patient can separate them only to a limited extent. Chemosis is formed at the margin of the cornea by reason of the anatomical relations of the conjunctiva. The conjunctiva is intimately and very closely connected with the cornea, and almost lost, as it spreads out on its surface. Over the schlerotic coat it is loosely attached by intervening connective tissue. The palpebral conjunctiva is thicker in substance, and its connection with the lid renders the part susceptible of edamatous swelling.

The reflected portion of the conjunctiva intervening between the lids and the eye-ball isvery loosely connected. Under gonorrhœal infiammation this an atomical relation of the conjunctiva has a very important effect upon the production of chemosis—a pathological condition about which a great deal has been said and written, and much recommended to be done, by oculists and surgeons, for the relief.

It is the result of infiltration,and is developed in this wise. The specific gonorrhœal pus, having come in contact with the conjunctiva, finds a fertile field for its implantation, and prolific reproduction; acting as a powerful irritant, it invites the nutrient blood to the parts, and all the conditions seem to exist to favor the active morbific changes that so rapidly follow. The specific virulent inflammation infiltrates the tissues; serum and plasma are thrown out into the meshes of the conjunctiva, and the connective tissue, filling the loose subcellular spaces beneath the reflected conjunctiva, it insinuates itself into the interspaces between the schlerotic and conjectiva, until the margin of the cornea is reached. Here the exudation stops ; there being no connective tissue or cellular spaces to be occupied. The conjunctiva, however, being forced forward by the accumulating exudation from beneath and behind, this membrane is forced to lap, or fold over and upon the margin of the cornea. This fold of the conjunctiva, in its altered condition, is what oculists talk and write so much about—the "choking of the corneal circulation;" and which causes ulceration, sloughing and destruction of the cornea, resulting in total blindness.

It is very apparent in this folding of the conjunctiva, its vessels and capillaries are bent on themselves,their calibre lessens, and their carrying capacity reduced. Is it conclusive to suppose that this state of the restricted circulation to the cornea, necessitates its destruction without very material aid from some other destructive force? How frequently do surgeons in operating upon the eye, cut away or disturb a large portion of the ocular conjunctiva without destruction of the cornea? And again, do surgeons not frequently sever with Beer's cataract knife, one half of the circumference of the corneal vascular connection with both the schlerotic and conjunctiva,without its destruction ?

The chemosis is *not* the essential cause of the corneal destruction. The conjunctival vessels do *not* afford the only source of vascular supply. Vessels pass from the schlerotic to the cornea. The schlerotic is not non-vascular tissue. The cause of the corneal destruction lies behind chemosis, with its mechanical effects upon the circulation. It consists of, and is due in part to, the metamorphic action of inflammation upon the tissues. It is also largely due to the local effect of the products of this inflammation, the contact of gonorrhœal pus, not only as an excitant to continued inflammatory action,but,also,by its corroding, desolving and disintregrating effects upon the tissues with which it comes in direct contact. How often do you witness its corroding and scalding effects upon the cheek of the infected? If it has this effect upon the skin, what must its ravages be upon the delicate tissues of the eye, its chosen seat of metamorphic aggression? Every physician and surgeon is familiar with the ravages made upon the tissues by ordinary pus, how it burrows and bores its way through the tissues, even to the bones. This being the effect of ordinary pus, how much more destructive must gonorrhœal pus be, when every leucocyte is poisoned with infection?

When we take into account the pathological condition that obtains in an eye infected with gonorrhœal inflammation, and the favorable circumstances, and vantage ground occupied by the pus, we can better appreciate its evil effects. The infected eye is rendered exceedingly irritable, and sensative to light, which is attended with an involuntary effort to keep the lid hermetrically closed. The lids are tipped with cartilage, and bordered with the orbicular muscle, the stimulated muscular fiber of which, causes a complete closure, even to an overlapping of the edges of the lids. This condition affords a secure lodgement and retention of the pus. So completely are secretions incarcerated and pent up within the lids, that I have seen the fluid spurt out forcibly upon their separation.

At a very early period of my professional life, some 32 years ago, I was called to the country, in Kentucky, to see a little infant but a few days old, whose eyes were infected with this disease. The mother informed me that the child had not opened its eyes for three days. The orbital shape showed out prominently through the lids. After cleansing and bathing the lids in warm water, I separated them, and to my horror and great astonishment, the substance of each ball was extruded in one disorganized, purulent mass. At this impressible period of my professional life, this incident produced a vivid and lasting impression on my mind. With my then crude knowledge of pathology, I could not but believe the destruction of the eyes in this case, held some connection with the disorganizing effect of the pent up pus. The many years of experience and observation since that time, has not lead me to relinquish this impression, nor modify the therapeutic indications suggested by it.

The pus may not only be pent up and occupy the entire palpebral cavity, but the activity of the pathological changes are favored by the contact of diseased surfaces; and in case of chemosis, this morbid effect is reinforced by the conjunctiva being doubled upon itself, and folded over, like a belt, upon the cornea; in some instances covering the entire cornea,and extending to,and even between the lids, affording an additional lodgement, and retention of corroding pus, directly in contact with the cornea. It is precisely here, on the margin and surfaces of the cornea beneath this fold, that the destructive corneal changes take place. By securely opening the lids with a speculum, and raising this fold from the cornea, you may first observe these changes. The margin of the cornea may be ulcerated, or may have assumed a hazy, softened, washed-leather appearance, which may be due, in part, to the changes incident to inflammation; but frequently, more largely due to the softening, dissolving and disintegrationg effect of the pus retained too long in contact with the cornea.

You will often find the destructive changes have been too rapid to be the result, solely, of the ulcerative process. The residue of the corneal destruction will be seen mixed with the pus, or attached, and coming away in shreds, tow-like in character, showing the process has been a rapid one for corneal ulceration.

I am well apprised of the fact that the destructive changes above refered to, are ascribed to the choking of the conjunctiva vessels, cutting off the nutrition to the cornea, and that it sloughs like a part that had been constricted as by a ligature. This view has been held by high authority; but I think Whorton Jones has fully demonstrated the infeasibility of the argument, and exposed the absurdity and falsity of the therapeutics based upon this chemosis idea. The indication for treatment in gonorrhœal ophthalmia has been pretty well outlined already in the previous pages. If there is one therapeutic indication more rational and more effective than all others combined, it is this: to cleanse and disinfect the involved parts. In fact, without it, all other agencies will prove futile and abortive as to curative results. This cleansing and disinfecting of the parts, to be effective, must be thorough, complete and continued, embracing the entire palpebral cavity, and the conjunctiva in all its folds and reflections. To accomplish this, with the swollen and sensative state of the eye, will require a great deal of patience and perseverence, by both physician and patient. This cleansing of the eye can only be accomplished by the aid of a spring speculum to hold the lids well apart, and by the use of a smoothly working syringe, with an even, well-rounded nozzle, and by carrying it then well up behind the lids, and throwing a well-directed stream to all parts of the conjunctival cavity. To aid in cleansing the eye, some surgeons have recently suggested, and very wisely too, that the speculum, itself, be made a further means of irrigation, by having it made of silver, and hollow, perforated with numerous holes, and attached by a tube to an irrigating bottle to contain the necessary fluid to cleanse and disinfect the eye. This may be brought to bear with well regulated force, by elevating the bottle. The surgeon, however, would find it necessary to supplement such a speculum by the use of the syringe, or by a nozzle throwing a more direct stream connected with the same tube, and both, that the chemosis fold of the conjunctiva, overlaping the cornea, might be raised from the surface of the cornea, and thoroughly washed out. If the swelling is great, and the difficulty of opening the lids is considerable, canthoplasty should be performed early in the disease, to relieve tension ; but more particularly to give space and opportunity for more thoroughly washing out the eye. This operation should not be deferred on account of inflicting pain or deformity, when found at all necessary ; as any deformity resulting, can be corrected by an after operation. This washing process, to be effective, must be used as often as the pus accumulates, if it be every half hour, or every hour of the twenty-four of the day. The menstruum to cleanse the eye may be any mild detergent wash, the quantity, rather than the strength, should accomplish the work. The bi-chlorate of soda will be found a most excellent one, of the strength of about a drachm to a pint of luke-warm water, or a weak solution of liquid chlorinated soda; lime water or salt water answer well; or a very weak solution of the vaunted corrosive mercury may be used. The tissues of the eye will be found very sensitive to the action of corrosive mercury. Carbolic acid, well diluted, alone, or, in combination with some other agent, will be found useful. To check excessive suppuration, and tissue change, nothing will be found so beneficial as a weak solution of nitrate of silver. The strength of the solution should not exceed a grain to the ounce of pure water, and this strength may be found too severe. To be effective, it must be used immediately after the thorough cleansing, and before the speculum is removed; and it should be poured into the eye in sufficient quantity to reach the entire surface involved, before the solution is decomposed; pour at least a drachm into the eye, and if this quantity is whitened by too much precipitation, let it run out, and pour it in again. This application may be made from two to four times during the twenty-four hours; the solution should be used weak, but in quantity; and the eye-ball should be moved about so as to insure contact with the entire mucous surface. A few drops of such a solution, dropped into the eye, is so promptly decomposed, by the secretions of the eye, that it amounts to a pidling business, and is too homœopathic to be effective.

In the acute stage, to subdue the rapid tissue change, small cloths, large enough to more than cover the involved parts, should be kept constantly applied, taken out of ice-cold water, and changed every few minutes, or as often as rendered necessary by the heat. Should the chemosis be found prominent, and folding over the cornea, it should be raised, cleansed, and the nitrate of silver solution be made to reach the entire super-imposed surfaces. Should ulceration appear at the margin of the cornea, it should be carefully and delicately touched by a small silver probe, tipped with nitrate of silver. This application should be made with great care, so as not to involve more than the ulcerated surface, and not be made oftener than every second day, and to be omitted when the ulceration ceases to extend. The treatment,thus far proposed,is essentially local, for the reason I understand gonorrhœal ophthalmia to be essentially and specifically a local disease, and to be combated as such. I think it hardly worth taxing your time in suggesting such other general indications of treatment arising from such other accidental,or constitutional derangements and peculiarities that may exist in each individual case. If the patient's bowels have been well relieved, a narcotic at bed-time is requisite; to be effective, one should be chosen that does not constipate the bowels; ʒss to ʒi tincture of hyoscymus, in camphor mixture, may prove beneficial; otherwise opium or morphia may have to be resorted to. It is not easy to lay down dietetic rules suited to all cases. The diet should be such as is calculated to keep the patient's vital powers to the level of ordinary health. As much plain, good, nutritious food should be taken as the stomach can safely digest, and just such an amount of stimulus as will aid digestion, and maintain vigor of the circulation; quinine is often useful in proper doses. In other words the destructive processes of ulceration and sloughing going on in the eye, should be combated and guarded against as we would the same process in any other part of the body.

The more rational views entertained of inflammation as applied to both external and internal organs have worked a great change in the treatment of all inflammatory diseases, except that of the eye. From old prejudices, or from some inexplicable reason, not even to this late day, are these rational views and reformatory measures accepted as applied to this organ of sight. The value and importance of this organ, to human prosperity and happiness, may have conspired to bring down the potent agencies that belong to a barbaric period in medicine. Any one, who may choose to satisfy himself, need not read many even of modern authors on this disease, to catch up the old exploded antiphlogistic ideas of treatment and regimen in fighting and battling with this disease. Go back to thirty years ago,and read authors of that day and time touching this disease, or purulent ophthalmia, in any form, and it is truly appalling to contemplate the irrational, heroic treatment then in vogue; and yet scarcely an eye was saved from the ravages of this disease. Bloodletting, tartar-emetic, mercury and starvation were practiced to the greatest degree. One author speaks of the only eye that he knew saved from this disease was a young lady, who had been bled 70 oz. in three days; she had been mercurialized and tortured at the same time. Modern medicine wonders that she lived at all, with or without eyes.

In this day and time, blood letting is about played out. But the old antiphlogistic theory of inflammation with storm in the eye,to be combated by mercury,tartar-emetic and low diet,is not a thing of the past in the treatment of diseases of the eye, and especially in gonorrhœal ophthalmia. Every large community, where the infection is ripe, will confront you with evidences to the contrary, with several vacant, stareless eyes, monumental to this barbaric treatment.

I have no knowledge of any therapeutic property possessed by mercury, no influence exerted by it upon the human economy, whether in a pathological, or physiological state, that establishes an indication, or that warrants the specific action of mercury as a remedial measure in the treatment of gonorrhœal ophthalmia. I am not unaware of the fact that it has been, and is now, asserted and maintained that it is indicated as a curative measure, in relief of chemosis, in that it prevents plastic exudation, and promotes its absorbtion when exudated; that it aids in opening up the constricted circulation to the cornea. This is hardly tenable, since the present accepted view of inflammation is, that the exudation of plasma is often conservative, and is not one of the destructive elements of inflammation. That mercury has the property to reach around through the circulation to the constricted conjunctival vessels, and by some occult force, open up blood-vessels, mechanically obstructed, making a nutritious pathway to the cornea, is too theoretical and far-fetched to receive favor or credence. It is further claimed and stated that mercury is indicated in this disease, as a difibrinizer of the blood, as though fibrin was fuel to the fire of inflammation! This proposition need hardly be discussed, since it is physiologically established that fibrin, as such, does not exist in the blood. Well has it been stated "so true is it that the vulgar errors of to-day are but solemn dicta of our fore-fathers."

